# History Bias, Study Design, and the Unfulfilled Promise of Pay-for-Performance Policies in Health Care

**DOI:** 10.5888/pcd13.160133

**Published:** 2016-06-23

**Authors:** Huseyin Naci, Stephen B. Soumerai

**Affiliations:** Author Affiliation: Huseyin Naci, Department of Social Policy, London School of Economics and Political Science, London, United Kingdom.

**Editor’s Note**: In June 2015, *Preventing Chronic Disease* published “How Do You Know Which Health Care Effectiveness Research You Can Trust? A Guide to Study Design for the Perplexed,” which used simple graphs and easy-to-understand text — in 5 case studies — to illustrate how powerful biases, combined with weak study designs that cannot control for those biases, yielded untrustworthy findings on influenza vaccination policy, health information technology, drug safety, prevention of childhood obesity, and hospital safety (“mortality reduction”) programs. The target audiences for that article were policy makers, journalists, public health and medical trainees, and the general public; the primary goal was to explain how weak or strong study designs fail or succeed in controlling for biases. In the Editor’s Note, we promised to add to those examples of common biases and research designs to show why people should be cautious about accepting research results — results that may have profound and long-lasting effects on health policy or clinical practice, some of which could be detrimental to health.

In this sixth case study, we revisit one of the most common and dangerous threats to research validity: history bias (ie, researchers’ failure to consider relevant events or changes that precede an intervention or co-occur while it is in progress). Studies that fail to control for history can mislead policy makers and clinicians. The pay-for-performance policy used to illustrate history bias in this article is sensitive to this powerful bias, because medical practice is always changing as a result of factors unrelated to policy. Without investigating changes in a study’s hoped-for outcome over time both before and after the policy or intervention being studied is implemented, investigators will probably attribute those changes to effects of the policy they are studying, causing billions of dollars of waste implementing such policies worldwide.

## Introduction

The ongoing flip-flopping of research findings about the effects of medical or health policies weakens the credibility of health science among the general public, clinicians, members of Congress, and the National Institutes of Health (1–3). Even worse, poorly designed studies, combined with widespread reporting on those studies by the news media, can distort the decisions of policy makers, leading them to fund ineffective, costly, or even harmful policies. Several reports in top medical journals in 2015 (4–6) pronounced that economic incentives in Pioneer Accountable Care Organizations saved medical costs, but the reports did not control for major biases created by unfairly comparing selected high-performing organizations with less-experienced control organizations (7). The result? The US Centers for Medicare & Medicaid Services cited the findings as a reason for expanding the program nationwide.

Building on an earlier article in *Preventing Chronic Disease* (8), this article focuses on a widely accepted but questionably effective (9) health policy that compensates physicians for meeting certain quality-of-care standards, such as measuring or treating high blood pressure. Policy makers often believe that such financial incentives motivate physicians to improve their performance to maintain or increase their incomes, thereby improving patient outcomes (10). Health care systems in the United States, Canada, Germany, Israel, New Zealand, Taiwan, and the United Kingdom have committed billions of dollars to this approach in the hope that such incentives will improve the quality of health care (11). Although this monetary approach sounds good theoretically, international scientific reviews overwhelmingly find little evidence to support it (12). Giving physicians small incremental payments to do things they already do routinely (eg, measuring blood pressure) may be counterproductive and even insulting, may divert their attention from more critical concerns, and does not increase quality of care (13). Some studies even find that such compensation encourages unethical behavior by incentivizing doctors to “cherry-pick” healthy, active, wealthy patients over “costly” sick patients who are less likely to reach the performance targets. Nevertheless, this financial-incentive policy is entrenched in many components of the Patient Protection and Affordable Care Act (colloquially known as Obamacare), including Accountable Care Organizations, patient-centered medical homes, and health information technology (14).

In this article, our aim is to help the public and policy makers understand how a pervasive bias can undermine the results of poorly designed studies of pay-for performance programs published in even the world’s leading medical journals. We also point to observational study designs and systematic reviews of the total body of evidence to find more trustworthy conclusions on the efficacy of pay-for-performance (12). Although randomization is frequently not feasible for evaluating such public policies (15), we also present an example of a randomized controlled trial that supports the conclusions drawn from strong observational study designs.

## The Threat: History Bias

The most pervasive threat to the credibility of studies of pay-for-performance (and many other health interventions) is history bias. History biases are simple to understand: they are events unrelated to the policy under study that occur before or during the implementation of that policy and that may have a greater effect on the policy’s hoped-for outcome than the policy itself. These events call into question the conclusions of studies evaluating the policy. They can be any event, such as a concurrent improvement in physician practice that successfully identifies and treats patients with high blood pressure, or widespread news media coverage of a new drug or new national guidelines supporting a life-saving treatment (for example, β blockers to prevent acute myocardial infarction [16]). The American Heart Association’s physician educational campaign Get With the Guidelines led to a gradual improvement in hypertension management (17). If a study of a pay-for-performance program targeting hypertension management is launched after or in the middle of such a campaign and does not account for that campaign’s effect on any improvements, the pay-for-performance program may take credit for the success, even though the blood pressure improvements really resulted from the unmeasured historical changes in physician practices (18).

### Weak pre–post designs that did not control for history bias

Many studies have evaluated the United Kingdom’s pay-for-performance program — a national policy that provides financial incentives to physicians to improve patient care. The largest pay-for-performance program of its kind, this national policy, introduced in April 2004, offered family physicians up to an additional 25% of salary for meeting certain performance standards.

**Figure 1:**
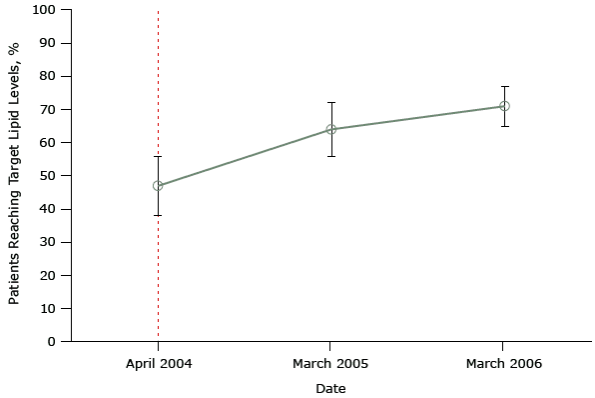
Mean percentage of patients achieving a selected quality indicator — a target total cholesterol level of ≤5 mmol/L— in a sample of family practices that participated in a study evaluating the effect of the United Kingdom’s pay-for-performance policy. Dashed line indicates when the pay-for-performance policy was implemented (April 2004). Figure is based on data extracted from Table 2 of Tahrani AA, McCarthy M, Godson J, Taylor S, Slater H, Capps N, et al. Diabetes care and the new GMS contract: the evidence for a whole county. Br J Gen Pract 2007;57(539):483–5 (19). **Table:** 

Figure 1 shows data from a study that did not protect against history bias when it evaluated the United Kingdom’s pay-for-performance program (19,20). The objective of this study was to determine whether the program led to improvements in a set of quality indicators — measurable elements of practice that indicate quality of care (in our example, target total cholesterol levels). This study had data only for the same month in which the policy was implemented and 2 points afterwards.

Month and Year	Percentage (Standard Deviation) of Patients Reaching Target Lipid Levels
April 2004	47 (9)
March 2005	64 (8)
March 2006	71 (6)

**Figure 2:**
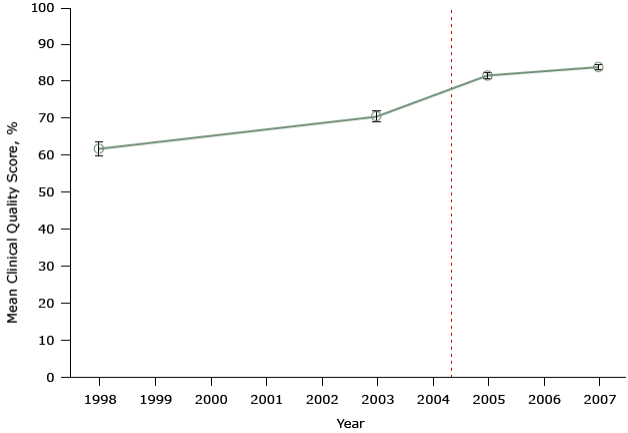
Mean clinical quality scores for diabetes at 42 practices participating in a study evaluating the effect of the United Kingdom’s pay-for-performance policy. The scale for scores ranges from 0% (no quality indicator was met for any patient) to 100% (all quality indicators were met for all patients). Dashed line indicates when the pay-for-performance policy was implemented (April 2004). Figure is based on data extracted from Table 1 in Campbell SM, Reeves D, Kontopantelis E, Sibbald B, Roland M. Effects of pay for performance on the quality of primary care in England. N Engl J Med 2009;361(4):368–78 (21). **Table:** 

Figure 2 shows data from another study that evaluated the United Kingdom’s pay-for-performance policy. This study also did not account for secular trends, yet it was published in a major medical journal and is highly cited (21). The authors’ assessment included only 2 points in time before and only 2 points after program implementation.

Year	Mean Clinical Quality Score	
	Percentage	Standard Error
1998	61.6	1.8
1999	No data	No data
2000	No data	No data
2001	No data	No data
2002	No data	No data
2003	70.4	1.5
2004	No data	No data
2005	81.4	0.8
2006	No data	No data
2007	83.7	0.7

The key problem with both of these studies, which purport to show the positive effects of the national pay-for-performance policy, is the use of only 2 data points during a long period before program implementation and 2 data points afterwards. From so few data, it is impossible to know what was happening to affect diabetes scores unrelated to the pay-for-performance policy before that policy was implemented. So we do not know if any small changes after policy implementation resulted from the pay-for-performance program or from some other changes in physicians’ practice. If anything, it appears that improvements before implementation (from 1998 to 2003) — to the extent these are detectable by examining only 2 data points — may have lessened or flattened, not increased after implementation of pay-for-performance. These studies are examples of a simple pre–post design. Only one or 2 observations (points) before and after pay-for-performance cannot control for secular trends (history) before program implementation. The pre-existing trajectory of good quality of care both before and after the intervention is unknown, and it is impossible to know whether the policy had any effect on this trend.

### Strong interrupted time-series design that controls for history bias

**Figure 3:**
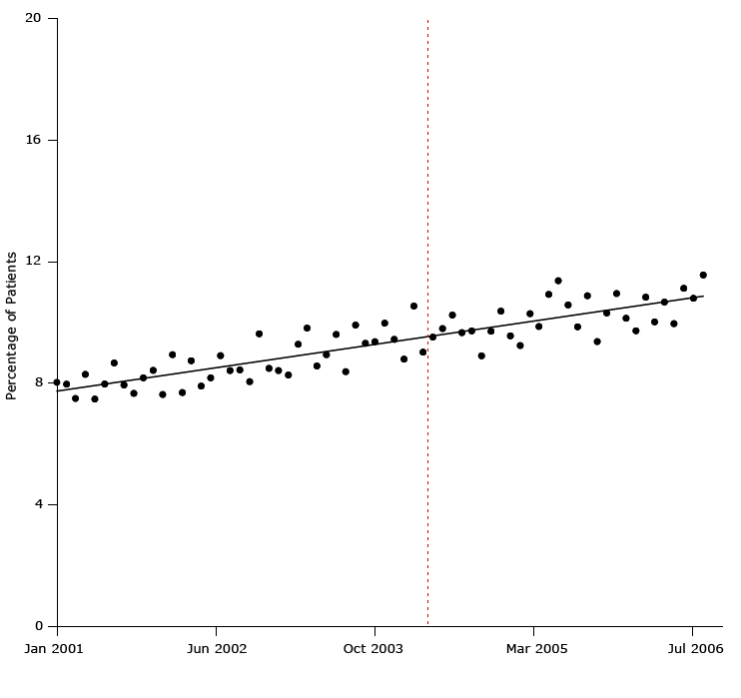
Percentage of study patients who began antihypertensive drug treatment from January 2001 through July 2006. Dashed line indicates when the United Kingdom’s pay-for-performance policy was implemented (April 2004). Figure is based on data extracted from bottom panel, Figure 3, in Serumaga B, Ross-Degnan D, Avery AJ, Elliott RA, Majumdar SR, Zhang F, et al. Effect of pay for performance on the management and outcomes of hypertension in the United Kingdom: interrupted time series study. BMJ 2011;342:d108 (22). **Table:** 

Figure 3 illustrates a result of one of the most convincingly negative studies showing that the United Kingdom’s pay-for-performance had no detectable effects on quality of care for patients with hypertension. Using a strong interrupted time-series design and 7 years of monthly data (84 time points) for 400,000 patients before and after the program’s implementation, Serumaga et al showed that the pay-for-performance program started in the middle of a slight rise in the percentage of patients who began blood pressure treatment (22).

Month and Year	Percentage of Patients
Jan-01	8.03
Feb-01	7.97
Mar-01	7.50
Apr-01	8.29
May-01	7.48
Jun-01	7.98
Jul-01	8.67
Aug-01	7.94
Sep-01	7.67
Oct-01	8.18
Nov-01	8.42
Dec-01	7.63
Jan-02	8.94
Feb-02	7.69
Mar-02	8.74
Apr-02	7.91
May-02	8.18
Jun-02	8.91
Jul-02	8.42
Aug-02	8.43
Sep-02	8.05
Oct-02	9.63
Nov-02	8.49
Dec-02	8.42
Jan-03	8.27
Feb-03	9.28
Mar-03	9.81
Apr-03	8.57
May-03	8.94
Jun-03	9.60
Jul-03	8.38
Aug-03	9.92
Sep-03	9.31
Oct-03	9.37
Nov-03	9.98
Dec-03	9.44
Jan-04	8.79
Feb-04	10.54
Mar-04	9.02
Apr-04	9.52
May-04	9.80
Jun-04	10.24
Jul-04	9.66
Aug-04	9.72
Sep-04	8.90
Oct-04	9.71
Nov-04	10.37
Dec-04	9.56
Jan-05	9.24
Feb-05	10.29
Mar-05	9.87
Apr-05	10.92
May-05	11.37
Jun-05	10.57
Jul-05	9.86
Aug-05	10.88
Sep-05	9.37
Oct-05	10.31
Nov-05	10.95
Dec-05	10.14
Jan-06	9.72
Feb-06	10.83
Mar-06	10.02
Apr-06	10.67
May-06	9.96
Jun-06	11.12
Jul-06	10.79
Aug-06	11.56

Every figure in the article shows flat or slightly improving treatment over many years and no effect of the $2 billion program that links family physician’s income to measures of health care quality. The existence of a long prepolicy trend, established by data for January 2001 through April 2004, to control for history bias (eg, pre-existing physician improvements in quality) enabled a valid assessment of the effect of the policy on changes in the level or trend in health outcomes over many years. The stronger study (interrupted time-series design) showed that the United Kingdom’s pay-for-performance program had no effects, whereas the 2 weak studies (pre–post design) contributed only to false or exaggerated hopes.

**Figure 4:**
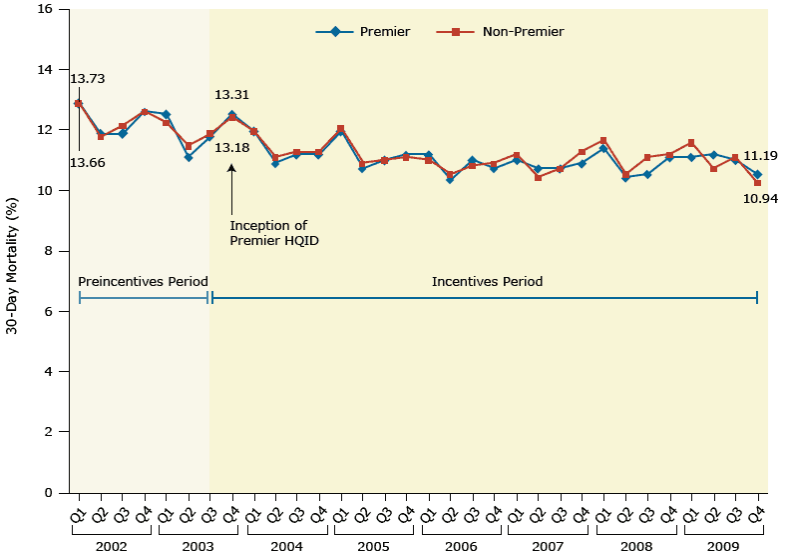
Mortality at 30 days among all hospitals examined before (from first quarter 2002) and after (through fourth quarter 2009) implementation of a pay-for-performance intervention (Premier Hospital Quality Incentives Demonstration [HQID]), which targeted 4 conditions beginning in late 2003: acute myocardial infarction, congestive heart failure, and pneumonia, and patients who underwent coronary artery bypass grafting. Changes at hospitals participating in the pay-for-performance intervention (Premier) were similar to changes at hospitals not participating (non-Premier) for all 4 conditions. Figure is reproduced from Jha AK, Joynt KE, Orav EJ, Epstein AM. The long-term effect of premier pay for performance on patient outcomes. N Engl J Med 2012;366(17):1606–15 with permission from the *New England Journal of Medicine* (23). **Table:** 

Figure 4 shows a remarkably similar negative result of paying hospitals for their measured performance. Hospital pay-for-performance programs and physician pay-for-performance programs are developed under similar assumptions: linking pay-for-performance with certain hospital outcomes are expected to motivate hospital leaders to meet targets to maintain or increase their incomes, thereby improving patient outcomes.

Quarter (Q) and Year	30-Day Mortality for All 4 Conditions Combined, Percentage	
	Premier	Non-Premier
Q1 2002	13.7	13.7
Q2 2002	12.6	12.5
Q3 2002	12.6	12.9
Q4 2002	13.4	13.4
Q1 2003	13.3	13.0
Q2 2003	11.8	12.2
Q3 2003	12.5	12.6
Q4 2003	13.3	13.2
Q1 2004	12.7	12.7
Q2 2004	11.6	11.8
Q3 2004	11.9	12.0
Q4 2004	11.9	12.0
Q1 2005	12.7	12.8
Q2 2005	11.4	11.6
Q3 2005	11.7	11.7
Q4 2005	11.9	11.8
Q1 2006	11.9	11.7
Q2 2006	11.0	11.2
Q3 2006	11.7	11.5
Q4 2006	11.4	11.6
Q1 2007	11.7	11.9
Q2 2007	11.4	11.1
Q3 2007	11.4	11.4
Q4 2007	11.6	12.0
Q1 2008	12.1	12.4
Q2 2008	11.1	11.2
Q3 2008	11.2	11.8
Q4 2008	11.8	11.9
Q1 2009	11.8	12.3
Q2 2009	11.9	11.4
Q3 2009	11.7	11.8
Q4 2009	11.2	10.9

A study by Jha et al (23) used an interrupted-time series design with a comparison series to investigate differences in patient mortality rates between hospitals participating in a pay-for-performance program and hospitals not participating. The study compared data for patients with one of 4 conditions: acute myocardial infarction, congestive heart failure, and pneumonia, and patients who underwent coronary artery bypass grafting. The 7-year trends in 30-day mortality for the pay-for-performance and non-pay-for-performance hospitals almost completely overlapped, leaving little doubt that the program had no detectable effect on long-term mortality (Figure 4).

It is worth noting, however, that hospitals in the pay-for-performance program opted into that program and could have already had better outcomes than non-participating hospitals before the pay-for-performance program began (ie, they were anticipating the financial rewards). But the equivalent trends and pre-program improvements in mortality for both study groups makes it less likely that this bias would have changed the conclusion.

Interestingly, a more recent study of pay-for-performance effects on 30-day in-hospital mortality rates among patients with pneumonia, heart failure, or acute myocardial infarction in one region of the United Kingdom was also compromised by already occurring declines in mortality rates and a lack of clear differences in mortality rates between the study and comparison groups (24). These short-term declines in mortality rates were not maintained in the long term (25).

### Strongest designs for controlling for history bias: randomized controlled trials

The strongest design for evaluating policies is a randomized controlled trial (RCT). In such study designs, random allocation of participants into intervention and control groups increases the likelihood that the only difference between the group receiving the pay-for-performance intervention and the control group (the one not participating in pay-for-performance) is the intervention itself. In a recent RCT, physicians randomized to a pay-for-performance intervention were eligible to receive up to $1,024 per patient who met target cholesterol levels, whereas physicians in the control groups received no economic incentives for achieving better outcomes (26).

**Figure 5:**
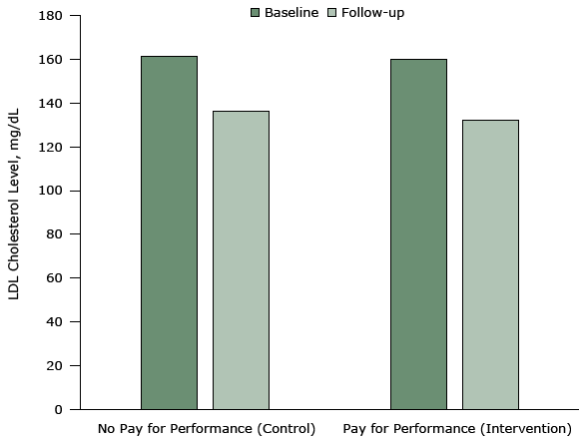
Mean low-density lipoprotein (LDL) cholesterol levels at baseline and 12-month follow-up in an intervention (pay-for-performance) group (in which incentives were provided to physicians) and a control group (no pay-for-performance). The intervention was conducted from 2011 to 2014 in 3 primary care practices in the northeastern United States. Patients in the control group achieved a mean reduction of 25.1 mg/dL in LDL cholesterol levels from a baseline of 161.5 mg/dL. Patients in the pay-for-performance group achieved a mean reduction of 27.9 mg/dL from a baseline of 159.9 mg/dL. The difference between the 2 groups was neither statistically significant nor clinically meaningful. Figure is based on data extracted from Asch DA, Troxel AB, Stewart WF, Sequist TD, Jones JB, Hirsch AG, et al. Effect of financial incentives to physicians, patients, or both on lipid levels: a randomized clinical trial. JAMA 2015;314(18):1926–35 (26). **Table:** 

Studies with strong, trustworthy designs, such as this RCT, suggest that paying physicians according to their measured performance on quality metrics (eg, reduction in low-density lipoprotein levels) does not improve outcomes (Figure 5). Physician payments did not produce any meaningful changes in quality of care compared with an equivalent group receiving no incentives.

Group	Low-Density Lipoprotein Cholesterol Level, mg/dL	
	Baseline	Follow-up
No Pay for Performance (Control)	161.5	136.4
Pay for Performance (Intervention)	159.9	132.0

### Rigorous systematic reviews of the entire body of pay-for-performance studies: the most trustworthy evidence

It is important to reiterate that no study is perfect, and no single study can determine the truth, whatever that may be. The accumulation of knowledge over time is the best way to assess a health care treatment or policy. However, given that most studies do not control for history or other biases, it is essential to single out the most rigorous systematic reviews — literature syntheses that eliminate weakly designed studies (the simple pre–post study designs illustrated in Figure 1 and Figure 2 or studies that simply correlate pay-for-performance with quality of care at one point in time) and summarize the remaining evidence.

One international systematic review (Box) (12) found that not only was there little evidence to support pay-for-performance’s effects on quality of medical care, some studies found that it sometimes had the unintended consequence of discouraging doctors from treating the sickest patients.

**Box. Conclusion of International Study by Houle et al in *Annals of Internal Medicine*, 2012: Does Performance-Based Remuneration for Individual Health Care Practitioners Affect Patient Care?: A Systematic Review (12)**

Although uncontrolled before–after studies suggested that P4P [pay for performance] improves adherence to quality-of-care indicators for chronic illnesses . . . higher-quality studies with contemporaneous control groups or analyses that considered secular trends failed to confirm these benefits. Most important, 4 large interrupted time series analyses conducted in the United Kingdom to evaluate the effect of their primary care P4P scheme introduced in 2004 found that quality scores for incentivized indicators were increasing for patients . . . before P4P began; there was no convincing evidence that the quality of care increased at a faster rate in the 3 years after P4P implementation than before.

[T]he current evidence for P4P targeting individual practitioners is insufficient to recommend wholesale adoption in health care systems at this time.

In addition to the international systematic review, other recent well-conducted systematic reviews supported the conclusion that questions the efficacy of pay-for-performance and advised against its widespread implementation — which has occurred despite the negative evidence (27). For example, Dutch researchers conducted an “umbrella review” — a review of all systematic reviews on pay-for-performance policies— to consider the totality of the evidence (28). They found that most systematic reviews unequivocally concluded that evidence showing effectiveness for pay-for-performance policies is weak, mixed, and inconclusive; many studies failed to find a meaningful effect attributable to the policy. As we have illustrated in this article, studies with weak designs that do not control for biases found more positive results than those with strong designs (29).

## Closing Comments

Despite its unfulfilled promise and discouraging evidence, this costly and ineffective approach to improving health care is a widespread component of current national and international health care policies. It is entrenched in many policies created by the Affordable Care Act (14). Part of the problem is the explosion in statistical techniques that attempt to “adjust for” or “correct” unquestionably dissimilar study and comparison groups rather than graphing the actual data over time so policy makers (who appreciate simple graphical displays) can actually look at the size of effects. Exaggeration of the effects of government programs through such “black box” statistics by the news media further widens the divide between reality and perception of policy effects (30). Weakly designed studies may also facilitate the proliferation of policies that encourage physicians to achieve “target rates” for health care procedures even when these procedures may be harmful for some patients. Oftentimes, what’s measured is what matters, and quality may deteriorate in areas that are not incentivized (31). Pay-for-performance programs may even result in collateral damage (13), diverting resources from under-resourced facilities such as safety net hospitals that provide care for vulnerable and high-need populations (32).

If we wish to encourage efficiency in medicine when our government and private health care programs are consuming almost one-fifth (17%) of the gross national product, it may be time to insist on strong experimental and quasi-experimental research designs (such as RCTs, interrupted time-series designs, and systematic reviews) in pilot tests of expensive policies. Investments of private and taxpayer funds should be based on solid evidence of safety and efficacy. The alternative, the present system, relies on weak and uncontrolled research designs, misleads policy makers and the public, and will ultimately lead to perverse effects, such as unsustainable costs, unhappy clinicians, and policies that may damage rather than improve the quality of medical care (30).
